# Regulation of autophagy by the PI3K-AKT pathway in *Astragalus membranaceus* -*Cornus officinalis* to ameliorate diabetic nephropathy

**DOI:** 10.3389/fphar.2025.1505637

**Published:** 2025-05-13

**Authors:** Rui Zhang, Xushan Lan, Wenhui Zhu, Lifan Wang, Peng Liu, Ping Li

**Affiliations:** ^1^ Xiyuan Hospital, China Academy of Traditional Chinese Medicine, Beijing, China; ^2^ Renal Division, Department of Medicine, Heilongjiang Academy of Chinese Medicine Sciences, Harbin, China; ^3^ College of Traditional Chinese Medicine, Changchun University of Chinese Medicine, Changchun, Jilin, China; ^4^ Shunyi Hospital, Beijing Hospital of Traditional Chinese Medicine, Beijing, China; ^5^ Beijing Key Lab for Immune-Mediated Inflammatory Diseases, China-Japan Friendship Hospital, Beijing, China

**Keywords:** diabetic nephropathy, *Astragalus membranaceus -Cornus officinalis*, calycosin, lipidomic technology, network analysis

## Abstract

**Aims and background:**

Autophagy plays an increasingly significant role in diabetic nephropathy (DN), but the mechanism by which autophagy participates in DN injury is not well understood. Our previous studies have shown that *Astragalus membranaceus - Cornus officinalis* (AM-CO) improves DN lipid metabolism disorders, however, the exact mechanism of which is also not well defined. The aim of this study was to investigate the therapeutic effects of AM-CO officinalis on DN and the mechanism of action on DN using lipidomic techniques and network pharmacological approaches.

**Experimental methods:**

The *in vivo* experiments were carried out using the KKAy mice model with the intervention of AM-CO. Analysis of kidney and serum samples from KKAy mice treated with AM-CO using lipidomic technology to obtain biomarkers for the treatment of DN and to identify the main targets associated with DN; Analyse potential signalling pathways for the treatment of DN using network pharmacology methods. *In vitro* experiments were performed with PA-induced HK-2 cells and results verified by protein blotting and immunofluorescence.

**Results:**

Lipidomic analysis revealed 363 differential metabolites in serum and 195 differential metabolites in kidney tissue, which were compared and analysed to find their common differential metabolites belonging to the phosphatidylethanolamine (PE) classes, respectively. In addition, PE plays a vital functiona in the process of autophagy. And the network analysis results speculated that Calycosin (Cal), a major component of AM-CO, could ameliorate DN injury by regulating autophagy through modulating the PI3K-AKT signaling pathway. *In vivo* experiments showed that AM-CO could induce autophagy, an increase in LC3II expression and a decrease in P62 expression. Meanwhile, *in vitro* experiments showed that Cal could also increase the expression of LC3II and inhibit the protein expression levels of p62, PI3K, P-AKT and AKT. The addition of a PI3K activator resulted in a reversal of protein expression.

**Conclusion:**

In conclusion, Cal can ameliorate the injury in DN by regulating autophagy, and PI3K-AKT is the main pathway for its regulation of autophagy and a key pathway for the action of AM-CO.

## 1 Introduction

Diabetic nephropathy (DN), a major complication of diabetes, is closely linked to the increasing global prevalence of diabetes (Alicic et al., 2017). Alongside a heightened incidence of cardiovascular disease and mortality, DN stands as the leading cause of end-stage renal disease, imposing a substantial global health burden and challenge (Selby and Taal, 2020). Current research has established that angiotensin-converting enzyme inhibitors and angiotensin receptor blockers are the primary therapeutic agents used to slow DN progression (Danfeng et al., 2024). However, despite their widespread use, treatment outcomes remain suboptimal, with some patients showing limited response. Due to the pathophysiological heterogeneity of DN and the variability in therapeutic efficacy, there is an urgency to identify novel therapeutic targets for DN management.

Autophagy, a vital physiological process, plays a crucial role in maintaining cellular homeostasis. Dysregulation of autophagy can lead to severe tissue damage (Parzych and Klionsky, 2014). Studies have shown that basal autophagy in renal cells—such as podocytes, proximal tubular epithelial cells, glomerular mesangial cells, and glomerular endothelial cells are essential for preserving the integrity and normal function of adult kidneys (Tang et al., 2020). Autophagy facilitates the clearance of damaged organelles and proteins accumulated under chronic cellular stress, thereby decelerating the progression of DN (Tseng et al., 2023). Under stressful conditions, the activation of autophagy has been demonstrated to exert renoprotective effects (Tan et al., 2018; Jaikumkao et al., 2024), Conversely, autophagy deficiency leads to mitochondrial damage, premature kidney aging, and fibrosis, ultimately increasing susceptibility to renal injury (Zheng et al., 2019; Liu et al., 2018). In recent years, numerous studies have explored the role of autophagy in elucidating DN pathogenesis and developing novel therapeutic strategies (Ma et al., 2020; Xiao et al., 2017). A growing body of evidence suggests that autophagy activity declines as DN progresses, establishing autophagy as a viable therapeutic target for DN intervention (Gonzalez et al., 2011).

Atg12 conjugation system and LC3 modification critically regulate autophagosome formation (Tanida et al., 2004). LC3-II serves as a key marker for autophagosomes, and after upon fusion of autophagosomes with lysosomes, LC3-II is degraded by lysosomal hydrolases (Kabeya et al., 2000). P62 is a multifunctional scaffold protein that plays a key role in regulating various signaling pathways and autophagy. It also serves as an adaptor protein, recognizing protein aggregates and specific organelles and directing them to autophagosomes for degradation. In this study, we primarily assess the occurrence of autophagy through the observation of LC3 and P62 proteins (Jeong et al., 2019; Kumar et al., 2022). PI3K signalling is a primary regulator of autophagy [18]. A downstream molecule of PI3K, AKT is critical for many cellular processes including apoptosis, cell survival, cell cycle progression, and metabolism (Huang et al., 2018). The PI3K-AKT pathway integrates signals from the cellular environment and other pathways, such as the MAPK/JNK and Ras/Raf/MEK/ERK pathways, thereby modulating the autophagic response (Wang et al., 2018; Zhou et al., 2015). Increasing evidence indicates that targeting the PI3K-AKT pathway to regulate autophagy could provide novel therapeutic avenues for disease treatment (Yang et al., 2018a; Wang et al., 2024).

*Astragalus membranaceus* (AM) and *Cornus officinalis* (CO) are two traditional Chinese botanical drugs recognized for their diverse pharmacological activities, including anti-inflammatory, anti-cancer, antioxidant, and anti-apoptotic effects (Ying et al., 2024; Wu et al., 2023; Jeff Yat-Fai et al., 2023). Our previous studies demonstrated the potential of *AM* and *CO* in alleviating lipid metabolism disorders in DN. However, it remains unclear whether these botanical drugs can delay DN progression via the autophagy pathway. The major metabolite of *Cornus officinalis*, Morroniside, has shown efficacy in improving DN in previous research (Zhu et al., 2023a),Thus, we selected Calycosin (Cal), a natural product of *AM*, for *in vitro* validation.

In this study, we investigated the impact of *AM* -*CO* on autophagy in KKay mice, while Cal was used for *in vitro* studies on PA-induced human proximal tubular cells (HK-2) to evaluate its effect on DN-related autophagy and associated pathways. Additionally, we applied lipidomics to analyze renal and serum samples from mice and employed network analysis to confirm whether Cal ameliorates DN progression by modulating autophagy through the PI3K-AKT pathway.

## 2 Materials and methods

### 2.1 Botanical drugs

AM (Leguminosae, Astragalus membranaceus (Fisch.) Bge.) and CO (Cornaceae, Cornus officinalis Sieb. et Zuce) were purchased from the Beijing Institute of Traditional Chinese Medicine, and their extraction process involves boiling in water. After boiling, the obtained medicinal solution is vacuum freeze-dried, ground into powder, and stored at low temperature in a well-ventilated environment until use. The weight ratio of AM-CO is determined in accordance with the composition and weight ratio within the Tangshen Formula (TSF).

Calycosin (with a chemical structure of C_16_H_12_O_5_, a molecular weight of 284.26, and a purity of over 98% as determined by high - performance liquid chromatography) was procured from Solarbio. The product comes in a 20 - mg package, with the item number SC8040.

### 2.2 Experimental animals

A total of 10 eight-week-old C57BL/6J mice and 20 KKAy mice (a DN mouse model) were selected for the animal experiments. All mice were housed under specific pathogen-free conditions with a 12-h light/dark cycle, a constant temperature of 25°C and 65% relative humidity. The KKAy mice were divided into two groups: the DN group, which was treated with saline, and the DN + AM-CO group, which received AM-CO solution via oral gavage (1.08 g/kg/day). AM - CO is derived from the TSF. In our previous research, the appropriate dosage of the TSF was found to be 2.4 g/kg/day (Wang et al., 2019; Kong et al., 2016). Given the composition of the TSF, in which the weight ratio of AM to CO is 10:3, through calculation, the dosage of AM - CO is determined to be 1.08 g/kg/day. The C57BL/6J mice, serving as the control group, received no treatment. In our study, we deliberately refrained from incorporating a positive control group. This strategic choice stemmed from the understanding that AM - CO is an essential and inseparable part of the TSF. Through our extensive and meticulous prior research, we have already conclusively demonstrated the remarkable efficacy of the TSF in mitigating kidney damage associated with diabetic nephropathy (Wang et al., 2024). All mice were acquired from VIEWSOLID Biotechnology Co. (Beijing, China) and were provided with tap water and a standard rodent diet for 2 weeks of acclimation. Following the acclimation period, the mice were returned to a normal diet and received the designated treatments for an additional 12 weeks. Blood and kidney tissue samples were collected after euthanasia for later analysis. The research protocol (No.: zryhyy21 - 22 - 01 - 09) has been approved by the Ethics Committee of the Sino - Japanese Friendship Research Institute of Clinical Medicine Science. Moreover, the experiment was carried out in strict accordance with the guidelines for the care and use of animals.

### 2.3 Lipidomics analysis

Kidney tissue and serum samples were collected from 5 C57BL/6J mice and 10 KKAy mice. For each sample, 50 mg of kidney tissue and 50 µl of serum were used. Quantitative lipidomics analysis was conducted using high-throughput LC-MS/MS (QTRAP 6500+, Sciex). The metabolomics data analysis encompassed both basic and advanced methods. Basic analyses included univariate statistical methods such as Student’s T-test and multivariate statistical methods such as Principal Component Analysis (PCA). Visualization and functional interpretation of differential metabolites were achieved through hierarchical clustering analysis and chord diagram analysis.

### 2.4 Drug targets of cal

To predict the potential targets of Cal, we utilized the SwissTarget Prediction tool. The structural and SMILES (Simplified Molecular Input Line Entry System) files of Cal were obtained from the PubChem database (https://pubchem.ncbi.nlm.nih.gov/). The SMILES file was then imported into SwissTarget Prediction to perform target prediction. SwissTarget Prediction is a small molecule protein target prediction tool that leverages reverse screening based on similarity principles, effectively predicting the most probable protein targets for a given small molecule.

### 2.5 Therapeutic targets for DN

Potential therapeutic targets for DN were identified using multiple databases, including DrugBank (https://go.drugbank.com), CTD (http://ctdbase.org), DisGeNET (https://www.disgenet.org), GeneCards (http://www.genecards.org), and OMIM (http://omim.org). Venn diagrams were used to visualize and select candidate targets for DN. These five databases integrate gene, phenotype, and disease information derived from literature-based and manually curated interactions, enabling insights into emerging information in human genetics.

### 2.6 Protein-protein interaction and bioinformatics analysis

Cytoscape 3.9.1 software (http://www.cytoscape.org/) was used to construct a protein-protein interaction (PPI) network of common targets. Venn diagrams were employed to identify overlapping genes between the compound and the disease. The shared targets were then imported into the STRING database (https://www.string-db.org/) to create a PPI network. The parameters were set as follows: “Organism” was selected as “*Homo sapiens*,” the confidence score was set to “medium confidence (0.400),” and isolated proteins were hidden from the network to obtain a clearer PPI network. The resulting network was further optimized using the NetworkAnalyzer plugin in Cytoscape, with node sizes adjusted according to the degree value of each gene. To further elucidate the biological functions of Cal in DN, gene ontology (GO) and Kyoto Encyclopedia of Genes and Genomes (KEGG) pathway enrichment analyses were used to investigate the overlapping genes. The common genes were then uploaded to the Metascape database (http://metascape.org/) for functional annotation and to the CnSKnowAll platform (https://cnsknowall.com/) for data visualization.

### 2.7 Histopathological analysis

The procedure for obtaining histological sections is as follows: Kidney tissues were first fixed in 4% paraformaldehyde, followed by paraffin embedding and cut into 4-mm thick sections. The specimens were then subjected to Hematoxylin and Eosin (HE) staining, Masson’s trichrome staining, and Periodic Acid-Schiff (PAS) staining. Renal pathological changes were assessed using a light microscope.

### 2.8 Cultivation of HK-2 cells

Human renal tubular epithelial cells (HK-2) were kindly provided by Professor HY Lan (The Chinese University of Hong Kong). The cells were cultivated in low-glucose DMEM medium (1.0 g/L; Corning Cat. No. 10-013, United States) with 10% fetal bovine serum (Thermo Fisher Scientific, Waltham, MA, United States) and 1% 1X penicillin-streptomycin (dual antibiotics). The culture conditions were controlled at 37 °C with 5% CO_2_ in a humidified incubator. To establish an autophagy impairment model mimicking DN, the HK-2 cells were induced with sodium palmitate (PA) (50 μM). The PA (from Sigma, United States) preparation requires weighing 27.8 mg of PA and adding it to 1 mL of deionized water. Heat the mixture to 70°C and check for dissolution and uniform mixing. Once completely dissolved into a clear solution, add 19 mL of culture medium containing 1% bovine serum albumin (BSA) and dilute to a final concentration of 5 mM. Subsequently, the cells were treated with Cal (5 μM, 10 μM, 20 μM; SC8040, Solarbio) and 740 Y-P (30 μM, Cat. No. HY-P0175) for 24 h.

### 2.9 Cell viability assay

HK-2 cells in the logarithmic growth phase were seeded in 96-well plates at a density of 1 × 10^4^ cells per well. The cells were then stimulated with different concentrations of Cal for 24, 48, and 72 h. At the corresponding time points, CCK-8 reagent (Mei5 Biotechnology, Beijing, China) was added to the wells and incubated for an additional 1–4 h. Cell viability was assessed following the instructions of the CCK-8 kit, and absorbance was measured at 450 nm using a microplate reader.

### 2.10 Immunofluorescence staining

Immunofluorescence staining was carried out on kidney tissues and HK-2 cells using the following primary antibodies: anti-LC3B (1:100, 2775S, CST), anti-SQSTM1/P62 (1:100, sc-48402, Santa Cruz Biotechnology), anti-phospho-Akt (1:100, 4060T, CST), and anti-phospho-PI3K (1:100, 17366S, CST). After primary antibody incubation, the samples were subsequently incubated with appropriate secondary antibodies—Alexa Fluor 488-conjugated goat anti-mouse IgG (1:800, GB25301, Servicebio) or Alexa Fluor 594-conjugated goat anti-rabbit IgG (1:800, GB28301, Servicebio)—at room temperature for 2 h in the dark. Nuclei were counterstained with DAPI (10 μg/ml) for 5 min while avoiding light exposure, and the samples were then mounted with 80% glycerol. Images were captured using an LSM800 confocal laser scanning microscope (Zeiss, Germany).

### 2.11 Western blot analysis

Radioimmunoprecipitation assay (RIPA) lysis buffer was used to extract proteins from kidney tissues and HK-2 cells. Proteins were separated by Tris-Glycine SDS-PAGE and transferred onto a polyvinylidene difluoride (PVDF) membrane. The duration of the membrane transfer was adjusted based on the size of the proteins. After blocking with 5% BSA for 1 h, the membranes were incubated with the primary antibodies at room temperature for 2 h or overnight at 4°C. The primary antibodies and their dilutions were as follows: anti-SQSTM1/P62 (1:1000, sc-48402, Santa Cruz Biotechnology), anti-LC3B (1:1000, 2775S, CST), anti-Akt (1:1000, 46910T, CST), anti-phospho-Akt (1:1000, 4060T, CST), anti-PI3K (1:1000, GB11525-100, Servicebio), and anti-phospho-PI3K (1:1000, 17366S, CST). The membranes were then washed five times with Tris-buffered saline with Tween-20 (TBST), each time for 5 min. After washing, the membranes were then incubated with the secondary antibodies (rabbit anti-IgG, 1:3000, GB23204, Servicebio; mouse anti-IgG, 1:3000, GB23301, Servicebio) for 25 min. The membranes were washed again with TBST, and protein bands were visualized using the iBright CL1000 imaging system (Thermo Fisher Scientific, Waltham, MA, United States).

### 2.12 Statistical analysis

The protein signals from the aforementioned experiments were quantified using ImageJ software. All data were visualized and analyzed using GraphPad Prism version 8.0 (GraphPad Prism Software, La Jolla, CA, United States). Statistical results are presented as mean ± SEM. Multiple comparisons were carried out using one-way analysis of variance (ANOVA) followed by Dunnett’s *t*-test. Differences were considered statistically significant when *P* < 0.05.

## 3 Results

### 3.1 Lipidomics analysis of biomarkers linking AM-CO to DN

**FIGURE 1:**
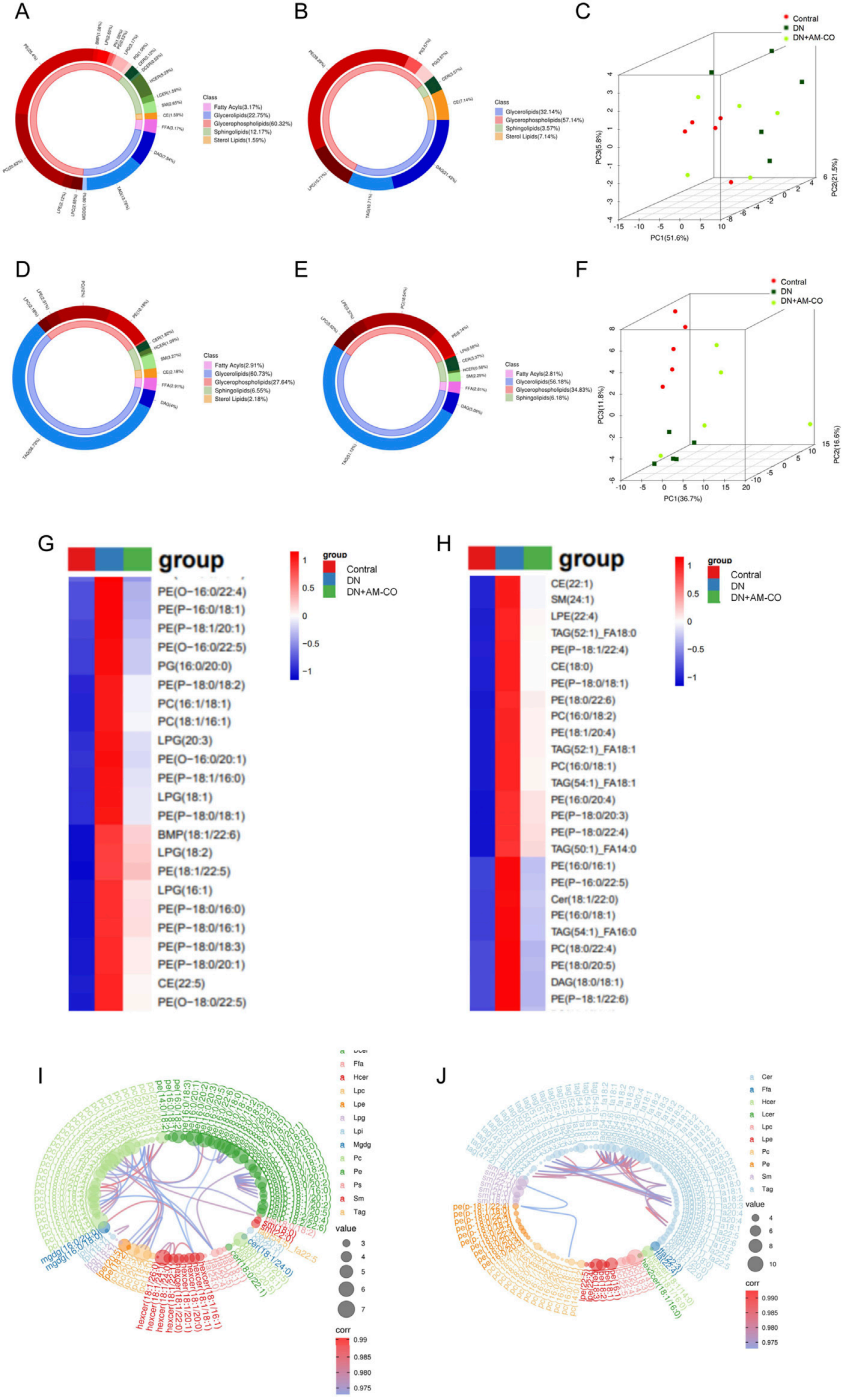
Lipidomic approach to the analysis of differential metabolites. **(A)** Metabolite classification and proportion of donut plot in control and DN groups (In kidney tissue); **(B)** Metabolite classification and proportion of donut plot in DN and DN+AM-CO groups (In kidney tissue); **(C)** Score scatter plot 3D of PCA model in kidney tissue; **(D)** Metabolite classification and proportion of donut plot in control and DN groups (In serum); **(E)** Metabolite classification and proportion of donut plot in DN and DN+AM-CO groups (In serum); **(F)** Score scatter plot 3D of PCA model in serum; **(G)** Heatmap of hierarchical clustering analysis in kidney tissue; **(H)** Heatmap of hierarchical clustering analysis in serum; **(I)** Chrodplot analysis in kidney tissue; **(J)** Chrodplot analysis in serum.

Circular diagrams were used to visualize the proportional composition of lipids in the analyzed samples (Figures 1A,B,D,E). The lipid composition analysis revealed that the major lipid classes in the samples included fatty acids, glycerolipids, glycerophospholipids, sphingolipids, and sterol lipids, with glycerophospholipids being the most abundant category. Principal component analysis (PCA) was performed on all samples to illustrate their distribution in an unsupervised manner. The 3D scatter plot of PCA displays the scores of the top three principal components, where different points represent distinct samples, and different colors and shapes denote various groups. Through PCA analysis, we observed significant differences in the lipid profiles across different groups (Figure 1C,F). The distribution of the normal group (Control) and the diabetic nephropathy (DN) group in the PCA plots showed clear distinction, indicating a substantial alteration in the lipid metabolic patterns in the DN group. After treatment with AM-CO, some improvement was observed, with samples closer to the control group. This suggests that AM-CO treatment plays a role in mitigating the metabolic disturbances induced by diabetic nephropathy. Hierarchical clustering analysis of differential metabolites and the corresponding heatmap (Figure 1G,H) demonstrated that after AM-CO intervention, there were 363 differential metabolites identified in serum and 195 in kidney tissue. Further comparative analysis revealed overlapping differential metabolites, predominantly belonging to the phosphatidylethanolamine (PE) class. Research has demonstrated that phosphatidylethanolamine (PE), with the assistance of the Atg5-Atg12-Atg16 complex, binds to soluble LC3, converting it into membrane-bound LC3-PE (Xu and Wan, 2023). Membrane-bound LC3-PE plays a crucial role in several key processes of autophagy, including the growth and expansion of the phagophore, the recruitment of cargo, and the fusion of autophagosomes with lysosomes (Kumar et al., 2018). This provides a basis for exploring diabetic nephropathy from the perspective of autophagy. Additionally, chord diagrams were used to provide a more intuitive visualization of the relationships and variations in lipid classification and content (Figures 1I,J). Through hierarchical clustering and chord analysis, we can further explore the relationship between lipid metabolism and renal pathological changes.

### 3.2 Network analysis and bioinformatics analysis of Cal’s therapeutic targets and pathways in DN

**FIGURE 2:**
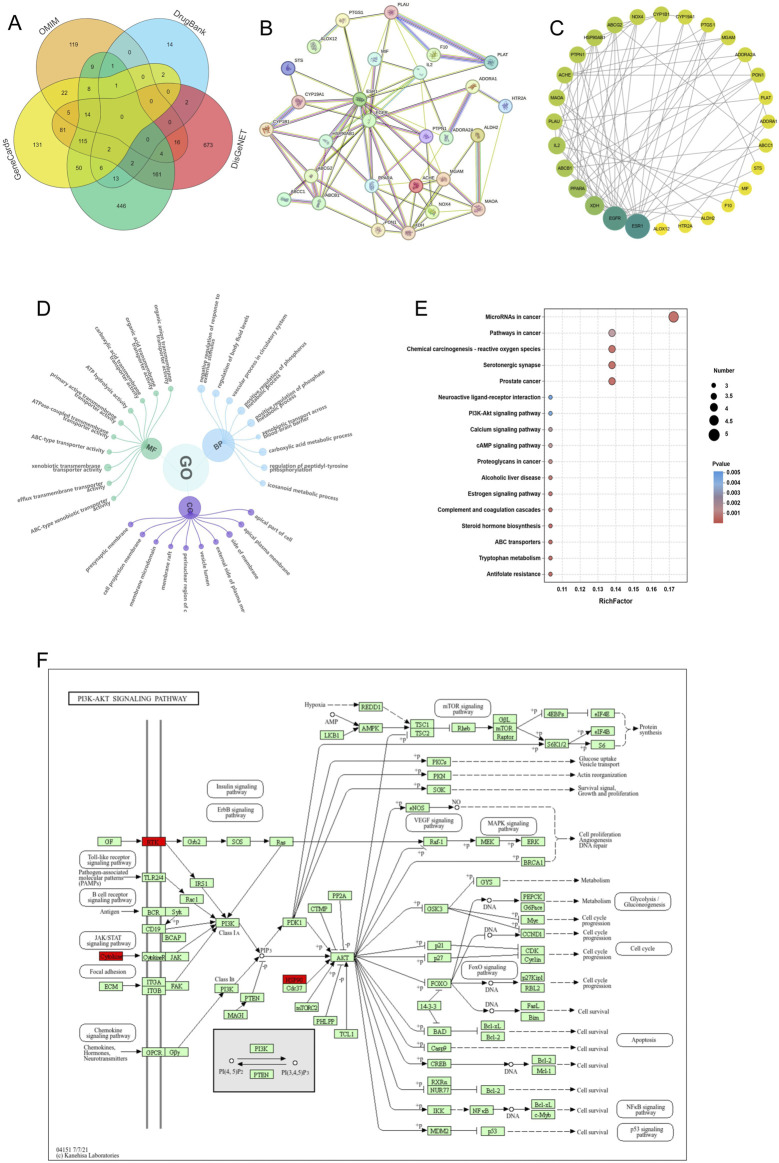
Network pharmacological analysis of Cal-related proteins for DN treatment. **(A)** Relevant targets of DN predicted by the database; **(B)** Target-gene interaction PPI network; **(C)** PPI network plot after combining Cytoscape; **(D)** GO enrichment analysis of predicted targets of Cal for DN; **(E)** KEGG analysis of predicted proteins of Cal for DN; **(F)** Pathway map of Cal‘s potential therapeutic targets for DN.

To gain a deeper understanding of the potential molecular targets of Cal (Cal) in DN management, we conducted a network analysis. First, we identified 52 predicted targets of Cal through database searches. Next, we retrieved 1,883 DN-related targets from multiple databases, including DrugBank, CTD, GeneCards, DisGeNET, and OMIM (http://omim.org) (Figure 2A). By intersecting the predicted Cal targets with the DN-related targets, we identified 29 common targets potentially involved in Cal’s therapeutic effect on DN. A protein-protein interaction (PPI) network was constructed for these overlapping proteins using the STRING database. After excluding unconnected proteins, the final network consisted of 29 nodes and 74 edges, with an average node degree of 5.1, an average local clustering coefficient of 0.498, and a PPI enrichment *p*-value of <1.0e-16 (Figure 2B). The resulting PPI network was then imported into Cytoscape for further analysis. Topological analysis of the PPI network indicated that ESR1 had the highest degree value (Figure 2C), suggesting that it may play a pivotal role in Cal’s therapeutic action.

Through bioinformatics analysis, we identified 305 significantly enriched Gene Ontology (GO) terms, including 237 biological processes (BP), 26 cellular components (CC), and 41 molecular functions (MF). The top 10 enriched terms for BP, CC, and MF with the highest gene counts were visualized (Figure 2D). Additionally, KEGG pathway analysis was conducted to predict the potential pathways through which Cal exerts its therapeutic effects in DN. We identified 18 statistically significant pathways, including chemical carcinogenesis - receptor activation, prostate cancer, antifolate resistance, PI3K-Akt signaling pathway, and neuroactive ligand-receptor interaction, among others (Figures 2E,F). Based on these findings, we selected the PI3K-Akt signaling pathway as our primary research focus. Earlier studies have reported that the inhibition of the PI3K-Akt pathway can induce apoptosis in cancer cells and promote protective autophagy (Rong et al., 2020). This supports the scientific basis and testability of exploring Cal’s therapeutic effects on DN by focusing on the PI3K-Akt pathway and autophagy regulation.

### 3.3 AM-CO improves physiological parameters and alleviates renal injury in DN

**FIGURE 3:**
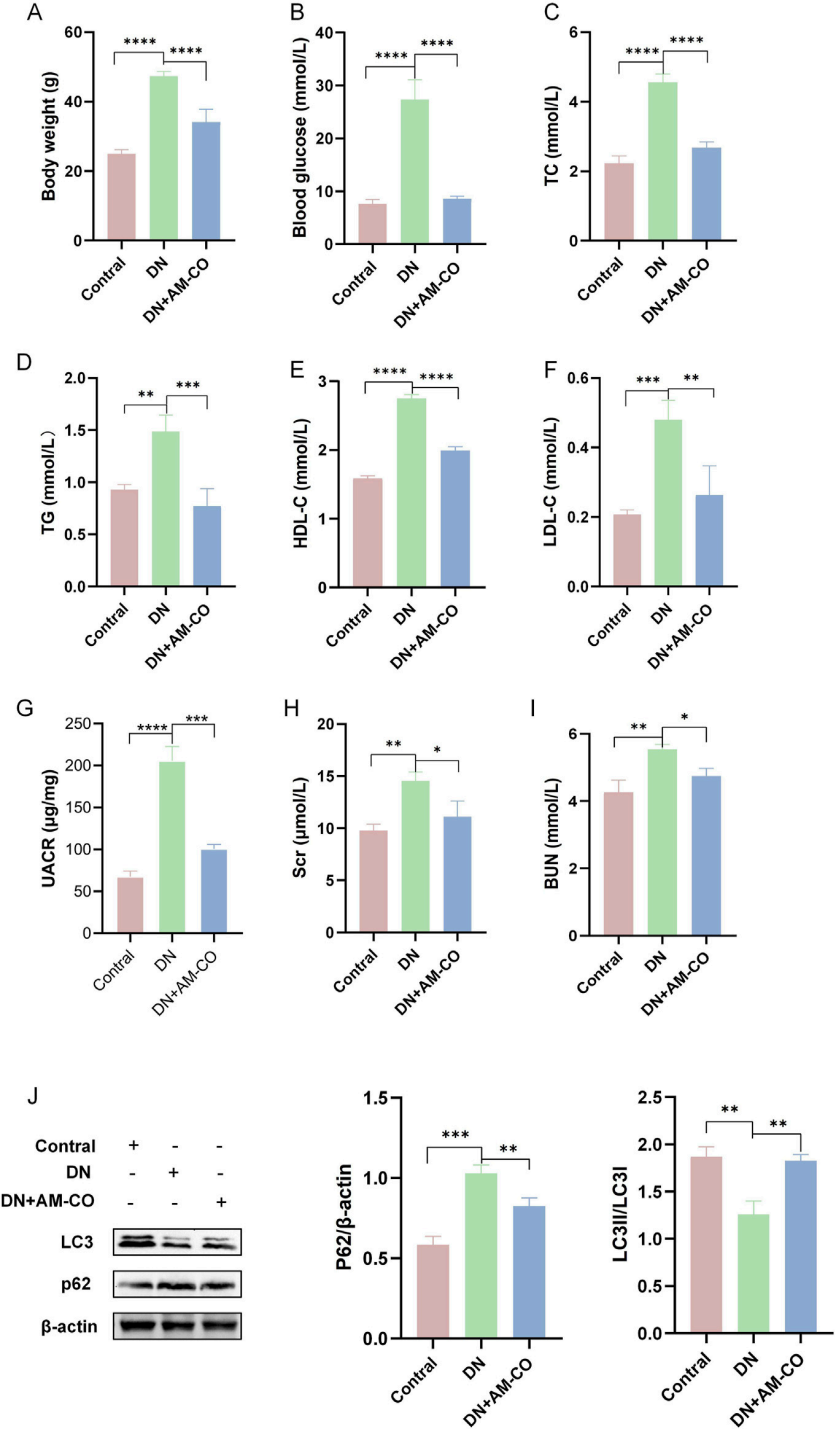
AM-CO attenuates abnormal physiological parameters and renal function in DN mice. **(A)** AM-CO reduced the level of body weight. **(B)** AM-CO reduced the level of blood glucose. **(C)** AM-CO decreased TC in blood; **(D)** AM-CO decreased TG in blood; **(E)** AM-CO decreased LDL-C in blood; **(F)** AM-CO decreased HDL-C in blood; **(G)** AM-CO decreased the level of UACR; **(H)** AM-CO AM-CO reduced the level of Scr in blood; **(I)** MOR decreased the level of BUN; **(J)** Western blot analysis demonstrates the expression of P62 and LC3. Data are expressed as mean ± S.D.; N = 10; *P < 0.05; **P < 0.01; ***P < 0.001, and ****P < 0.0001 compared with the DN group.

After 12 weeks of treatment, significant changes in physiological parameters were observed in DN mice (Figure 3). Compared to the model group, the AM-CO-treated group exhibited notable reductions in both body weight and blood glucose levels (Figures 3A,B). Furthermore, physiological parameters of blood lipids were substantially increased in the DN group, while AM-CO treatment effectively reversed the levels of TC, TG, HDL-C, and LDL-C (Figures 3C–F). Moreover, AM-CO demonstrated significant renoprotective effects. Serum creatinine (Scr), urinary albumin-to-creatinine ratio (UACR), and blood urea nitrogen (BUN) levels were markedly elevated in the DN group compared to the control group but showed significant improvement after AM-CO treatment (Figures 3G–I).

**FIGURE 4:**
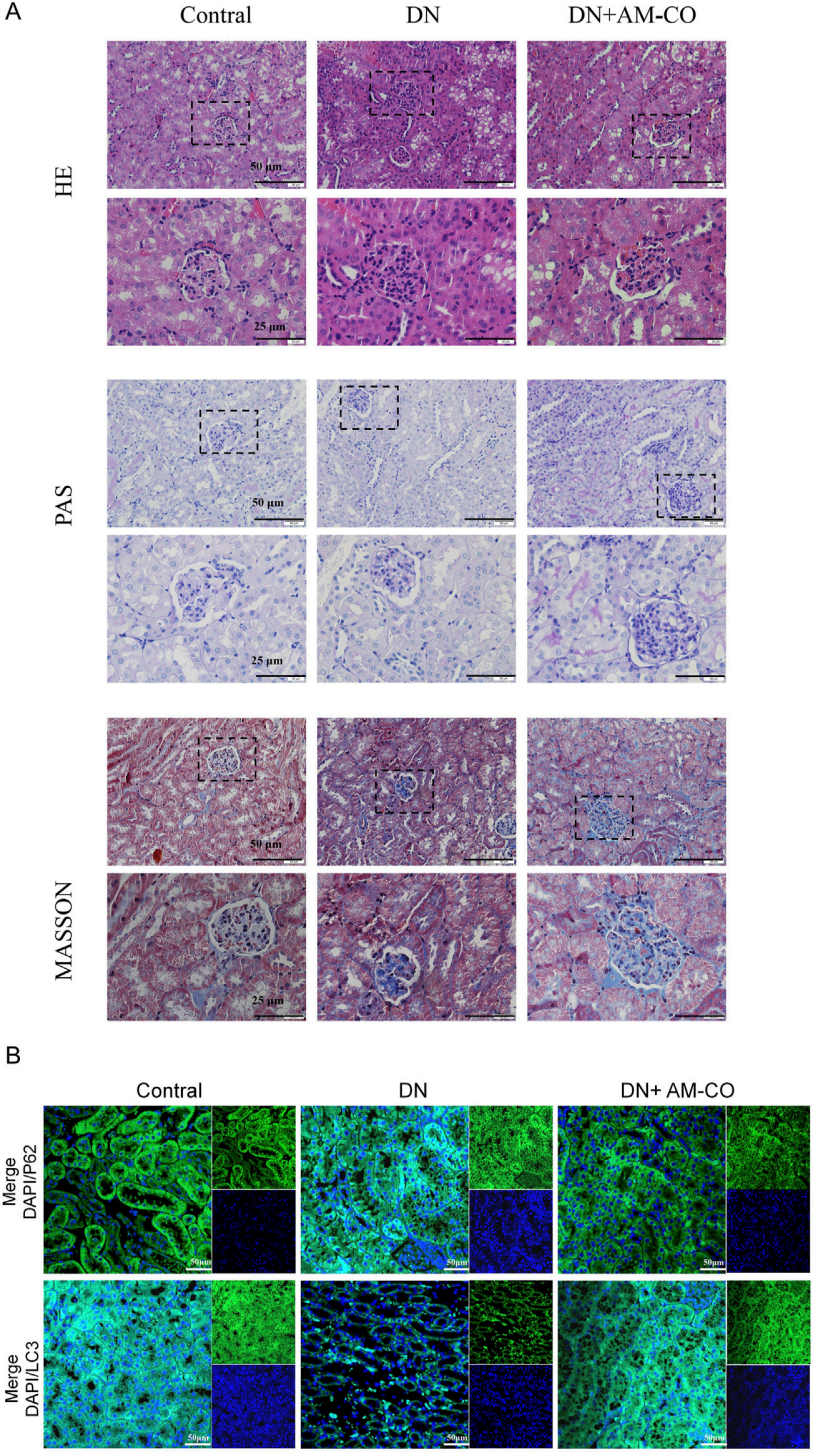
Effects of AM-CO treatment on renal pathology and autophagy in DN mice. **(A)** HE staining reacts to the degree of tubular dilatation, PAS staining shows the degree of glycoprotein accumulation, Masson staining the degree of collagen fibre deposition (scale bars: 25/50 μm). **(B)** Immunofluorescence staining showed expression of P62 and LC3 (scale bars: 50 μm).

To better evaluate renal pathological changes, we performed three types of staining: HE staining, PAS staining, and Masson’s trichrome staining (Figure 4A). HE staining revealed that the DN group exhibited severe renal injury characterized by tubular dilation. PAS staining indicated an increased accumulation of glycoproteins, while Masson’s staining showed a higher degree of collagen fiber deposition, indicative of aggravated renal fibrosis (Figure 4A). These pathological changes were significantly reversed in the AM-CO treatment group, suggesting that AM-CO has potent nephroprotective effects. Western blot analysis of renal tissue proteins showed that in the PA-induced DN model group, the expression of LC3II was decreased and the expression of P62 was elevated compared to the control group (Figure 3J). After AM-CO treatment, the expression levels of these two proteins were reversed, indicating that AM-CO enhanced autophagy and mitigated PA-induced renal damage. Immunofluorescence analysis further confirmed these findings. In the DN group, LC3II expression was reduced while P62 expression was increased. In contrast, the AM-CO and control groups showed high LC3II expression and low P62 expression, consistent with the Western blot results (Figure 4B). These results suggest that AM-CO enhances autophagy levels, thereby alleviating PA-induced renal injury.

### 3.4 Cal regulates the PI3K-Akt pathway to enhance autophagy in PA-inhibited HK-2 cells

**FIGURE 5:**
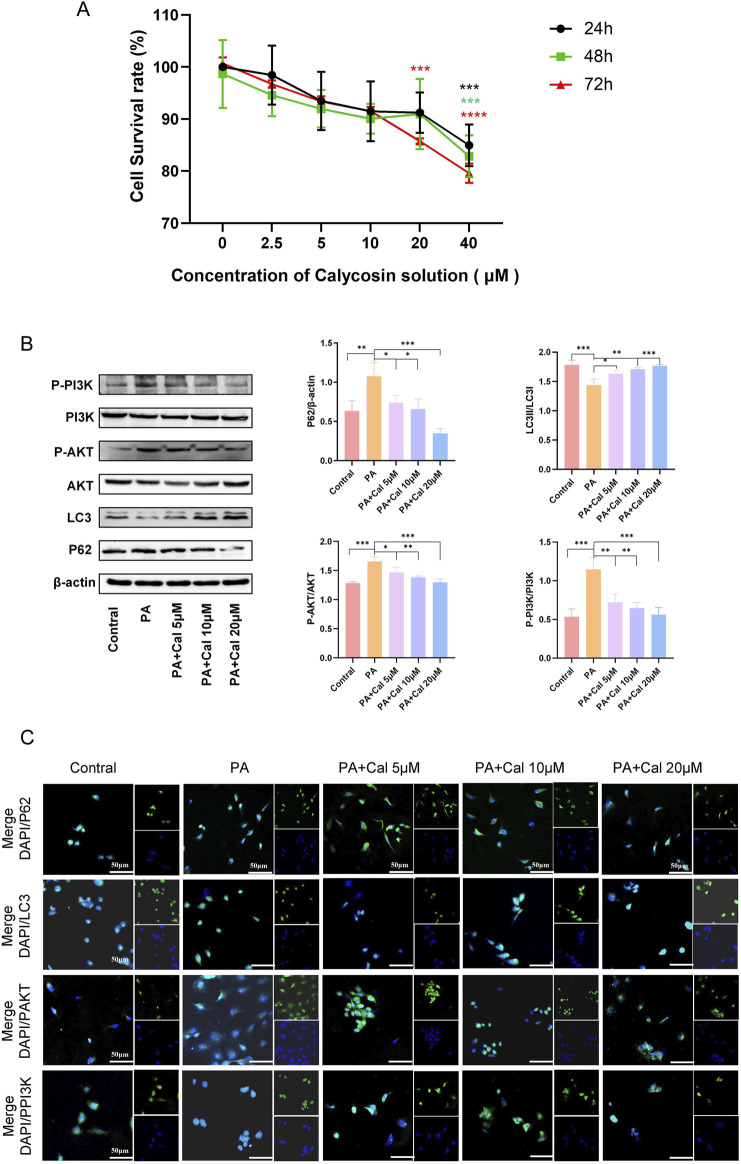
Effects of different concentrations of Cal on HK-2 cell viability and autophagy. **(A)** Effect of different concentrations of Cal on HK-2 cell viability; **(B)** Western blot showed the expression of P62, LC3, PAKT, PPI3K when different concentrations of Cal (5 μM, 10 μM, 20 μM) were given under PA stimulation; **(C)** Immunofluorescence results of P62, LC3, PAKT, PPI3K at different administration concentrations (scale bars: 50 μm). Compared to PA group, *P < 0.05, **P < 0.01, ***P < 0.001 and ****P < 0.0001.

The effect of Cal on the viability of HK-2 cells and its potential cytotoxicity was assessed using a CCK-8 assay. The results indicated that at a concentration of 40 μM, Cal exhibited significant cytotoxic effects (Figure 5A). In subsequent Western blot analyses, PA induction led to damage in HK-2 cells, as evidenced by a reduction in LC3II expression and an increase in P62 expression (Figure 5B). Treatment with Cal reversed these protein expression levels, indicating that Cal can effectively modulate autophagy. Furthermore, PA induction elevated the levels of phosphorylated AKT and PI3K proteins in HK-2 cells. Following Cal treatment, the levels of P-AKT and P-PI3K gradually decreased, showing a dose-dependent effect. As the concentration of Cal increased, the changes in protein expression became more pronounced (Figure 5B). The immunofluorescence results were consistent with the findings from the Western blot analysis (Figure 5C). LC3-positive areas were reduced following PA induction but showed a significant increase after Cal treatment. In contrast, the staining intensity of P62, P-AKT, and P-PI3K were notably higher under PA induction and decreased after Cal treatment, with a more pronounced reduction observed at higher Cal concentrations. These results suggest that Cal regulates the PI3K-Akt pathway to enhance autophagy in PA-inhibited HK-2 cells, thereby mitigating cellular damage.

### 3.5 Activation of PI3K reverses the therapeutic effects of cal

**FIGURE 6:**
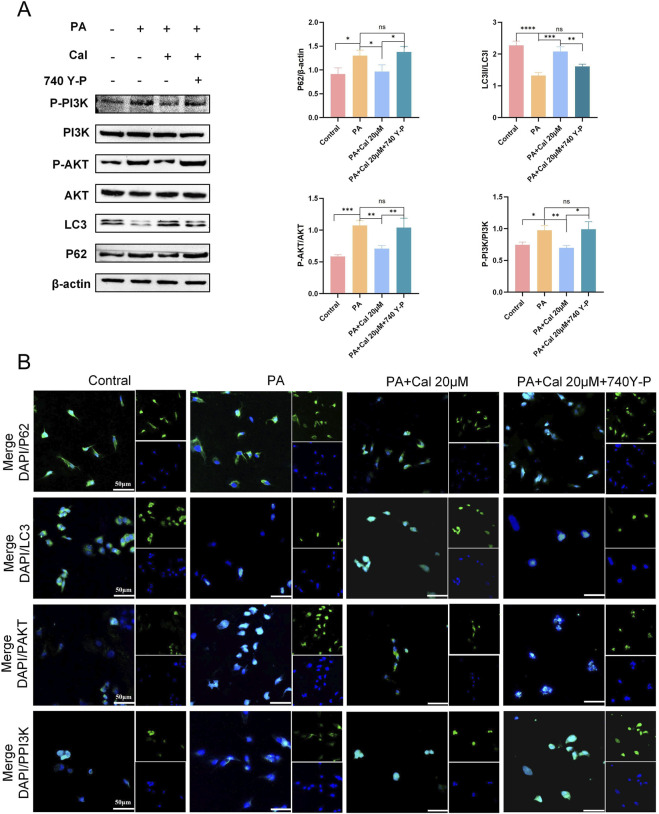
Addition of PI3K activators (740 Y-P) reverses Cal regulation of autophagy. **(A)** Protein expression levels of P62, LC3, PAKT, PPI3K after 740 Y-P treatment. Compared to PA group, *P < 0.05, **P < 0.01, ***P < 0.001 and ****P < 0.0001. **(B)** Immunofluorescence results of P62, LC3, PAKT, PPI3K after 740 Y-P treatment (scale bars: 50 μm). Compared to PA group, *P < 0.05, **P < 0.01, ***P < 0.001, ****P < 0.0001, ns:P > 0.05.

To further elucidate the mechanism by which Cal enhances autophagy and ameliorates DN, we utilized a PI3K activator, 740 Y-P, in conjunction with Western blot analysis. The concentration of 740 Y-P was set at 30 μM. The results demonstrated that the therapeutic effects of Cal were reversed upon treatment with 740 Y-P (Figure 6A). Specifically, LC3 expression decreased, while the expression levels of P62, P-AKT, and P-PI3K were elevated. The findings were further validated by comparing the 740 Y-P treatment group with the PA-induced group, showing no statistically significant difference between them. This indicates that the addition of 740 Y-P effectively nullified the effects of Cal treatment. Immunofluorescence has once again confirmed our results (Figure 6B). Consequently, we concluded that Cal exerts its therapeutic effect in DN primarily through modulation of the PI3K-Akt pathway to regulate autophagy.

## 4 Discussion

DN, one of the most frequent and life-threatening complications of diabetes, is a major cause of chronic kidney disease and renal failure (Chang and Hsu, 2018). The development of DN involves a complex interplay of various mechanisms, including cellular injury (Jin et al., 2023),inflammation (Tang et al., 2015), renal fibrosis (Yang et al., 2019),oxidative stress (Darenskaya et al., 2023), etc. Dysregulation of autophagy is also considered an important factor in the progression of DN. Autophagy is a cellular process that recycles nutrients, removes damaged organelles and protein aggregates, and eliminates invading pathogens. It plays a crucial role in development, physiology, aging, and disease in various organ systems, including the kidneys. Renal fibrosis, the final pathway of chronic kidney disease, has been increasingly associated with autophagy levels in various organs (Holvoet et al., 2017), Abnormal autophagy may be an important contributor to the pathogenesis of fibrosis (Mao and Fan, 2015). From the perspective of fibrosis, it becomes evident that autophagy plays a critical role in DN. In a streptozotocin-induced mouse model, activation of transcription factor 4 was shown to ameliorate renal fibrosis and reduce kidney injury by restoring autophagy (Liang et al., 2021). Our findings are in line with these observations, as both *in vivo* and *in vitro* experiments demonstrated the influence of DN on autophagy. Treatment with AM-CO and Cal effectively restored autophagy levels, achieving therapeutic benefits in the DN model. These results underscore the potential of targeting autophagy as a therapeutic strategy for DN management. In addition, in our study, treatment with AM-CO resulted in a significant improvement in blood glucose levels in DN mice. This observation suggests a potential association with glucose metabolism. Our previous research has shown that TSF can significantly reduce the blood sugar level of diabetes rats. Therefore, glucose metabolism may be our future exploration point.

**FIGURE 7:**
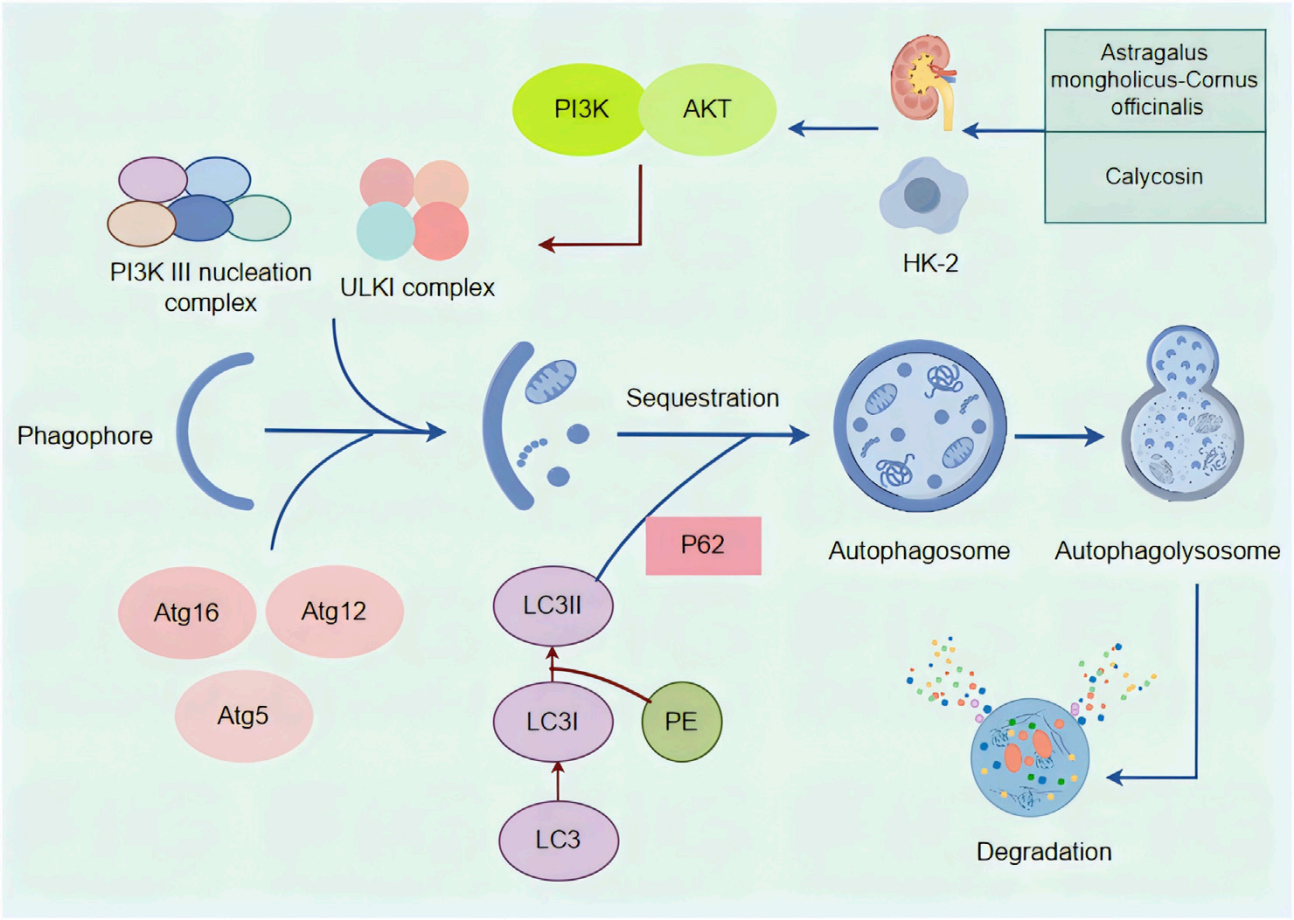
A mechanistic map of the formation and regulation of autophagy and the regulation of autophagy by *Astragalus membranaceus -Cornus officinalis* and Calycosin. The ULKI complex and PI3K III nucleation complex initiate autophagy, and the downstream Atg5-Atg12-Atg16 complex can turn PE-coupled LC3I into lipidated LC3II. P62 functions as an autophagy receptor and binds to LC3 to complete autophagy fusion and amplification. PI3K acts on AKT, which can directly act on ULKI complexes to regulate autophagy downstream. *Astragalus membranaceus -Cornus officinalis* as well as Calycosin can improve DN by modulating autophagy.

Autophagy is a multistep process involving initiation, nucleation, elongation, fusion, and degradation. The activation of autophagy is primarily regulated by a complex consisting of serine/threonine protein kinases ULK1, ULK2, and other proteins (Park et al., 2016; Zachari and Ganley, 2017), while the PI3K complex controls vesicle nucleation and phagophore formation (Zhong et al., 2009). Downstream of ULK1 and the PI3K complex is the ubiquitin-like Atg5-Atg12-Atg16 complex, which helps attach phosphatidylethanolamine (PE) to LC3. This process generates lipidated LC3, which becomes part of the autophagosome membrane and plays a key role in recruiting cargo. It also regulates the fusion and elongation of autophagosomes (Lin, 2017). P62, the first autophagy receptor identified in mammals, helps recognize cargo and deliver it to autophagosomes for lysosomal degradation. This mechanism is elucidated with remarkable clarity and precision in Figure 7.

The regulation of autophagy primarily involves three key pathways: the mammalian target of rapamycin complex 1 (mTORC1) pathway (Wan et al., 2018), the Sirtuin 1 (SIRT1) pathway, which is dependent on nicotinamide adenine dinucleotide (Xu et al., 2020), and the AMP-activated protein kinase (AMPK) pathway (Hoyer-Hansen and Jaattela, 2007; Kolahdouzmohammadi et al., 2023). Akt is phosphorylated and activated following the activation of PI3K. Once activated, Akt regulates processes such as cell proliferation, division, and metabolism through a cascade of downstream effectors, which in turn indirectly activate mTORC1. This activation subsequently inhibits both apoptosis and autophagy (Rajendran et al., 2024). The PI3K-Akt pathway, activated by growth factors, can inhibit autophagy independently of the mTOR pathway (Lipinski et al., 2010). Additionally, Akt can inactivate TSC2 (which inhibits mTOR phosphorylation) or suppress FoxO3 (a transcription factor that positively regulates autophagy), thereby inhibiting autophagy. In addition, when cellular energy is abundant, the PI3K-Akt pathway activates mTORC1 to inhibit autophagy. Conversely, under conditions of energy deprivation, AMPK promotes autophagy by inhibiting mTORC1 (Kma and Baruah, 2022). Additionally, SIRT1 regulates autophagy through deacetylation, thereby restoring cellular energy balance (Sun et al., 2023). Our study indicates that Cal can restore autophagy through the PI3K-Akt pathway, as demonstrated by increased phosphorylation levels of PI3K and AKT in the PA-induced model group, which were reduced following Cal treatment.

In our study, lipidomics analysis was first applied to assess kidney and serum samples from the DN model mice. Through differential metabolite comparisons, we identified PE as the most significant differential metabolite. As described earlier, PE can be conjugated to LC3 under the action of the ubiquitin-like Atg5-Atg12-Atg16 complex, generating lipidated LC3 that facilitates autophagosome fusion and elongation, playing a vital role in autophagy. This finding strengthens the rationale for targeting autophagy as a therapeutic approach for DN. Given that our previous research confirmed the therapeutic effect of morroniside on DN (Chen et al., 2024; Zhu et al., 2023b),we chose Cal as the focus of our *in vitro* study. Network analysis and bioinformatics results further revealed that Cal is significantly enriched in the PI3K-Akt pathway. Synthesizing these results, we explored the role of autophagy in DN through the lens of the PI3K-Akt pathway.

HK-2 cells are a human proximal tubular epithelial cell line commonly used to simulate tubular injury in DN (Garmaa et al., 2024). Based on *in vivo* and *in vitro* experimental results, Cal was found to ameliorate DN damage by regulating autophagy, with the PI3K-Akt pathway serving as its primary mechanism of action. The addition of 740 Y-P further validated the interaction between PI3K and Cal, providing robust evidence for our conclusion. Changes in LC3 and P62 expression levels also indicated PA-induced damage in HK-2 cells and the restorative effects of Cal on autophagy. Immunofluorescence, network analysis, and Western blot results were all consistent. Studies reporting that apigenin induces apoptosis and autophagy by inhibiting the PI3K/Akt/mTOR pathway in hepatocellular carcinoma are aligned with our findings (Yang et al., 2018b). However, this also highlights a limitation of our research, as we did not establish a direct link between the PI3K-Akt and mTOR pathways. As a result, it is unclear whether Cal exerts its effects on autophagy through the mTOR pathway.

In summary, our study demonstrates that Cal can mitigate DN damage by modulating autophagy, with the PI3K-Akt pathway being the primary mechanism involved and a key pathway for the therapeutic effects of AM-CO.

## Data Availability

The raw data supporting the conclusions of this article will be made available by the authors, without undue reservation.
